# Cold Disinfestation for ‘Red Globe’ Grape (Rhamnales: Vitaceae) Infested With *Drosophila suzukii* (Diptera: Drosophilidae)

**DOI:** 10.1093/jisesa/ieaa043

**Published:** 2020-06-01

**Authors:** Xiaoxue Wang, Guoping Zhan, Lili Ren, Shuangyan Sun, Haiyan Dang, Yifan Zhai, Hong Yin, Zhihong Li, Bo Liu

**Affiliations:** 1 Chinese Academy of Inspection and Quarantine, Beijing, China; 2 Department of Entomology, College of Plant Protection, China Agricultural University, Beijing, China; 3 Research Center for Standards and Technical Regulations, General Administration of Customs, Beijing, China; 4 Taiyuan Customs, Shanxi, China; 5 Institute of Plant Protection, Shandong Academy of Agricultural Sciences, Shandong, China

**Keywords:** spotted wing drosophila, cold treatment, phytosanitary treatment, cold tolerance

## Abstract

The spotted wing drosophila, *Drosophila suzukii* Matsumura, which is widely spread in the main soft-skinned fruits production areas in China, presents a threat to importing countries. In order to develop a phytosanitary cold treatment measure for preventing the movement of this drosophila fly, cold tolerance of six immature life stages of *D. suzukii* was compared followed by time-mortality and large-scale confirmatory tests on the most tolerant stage in grape fruit. Egg was defined as the most cold-tolerant stage by comparing the mortality of all the immature stages (egg, first, second, and third instars, early and late pupa) treated at 0 and 2°C. The minimal lethal time (LT) for 99.9968% mortality (95% confidence level [CL]) estimated by the probit model was 10.47 d at 0°C and 11.92 d at 2°C, respectively. Hence, 11 d (at 0°C) and 12 d (at 2°C) were chosen as the target time to conduct the confirmatory tests. No survivors were found among the estimated 50,385 and 57,366 treated eggs, which resulted in the efficacy of 99.9941 and 99.9948% mortality (95% CL) at 0 and 2°C, respectively. Our study suggests a technical basis for cold disinfestation on *D. suzukii* in cage-infested Chinese ‘Red Globe’ (*Vitis vinifera* L.) grape, which could provide flexible phytosanitary treatment for control of *D. suzukii* in the international trade of grape.

The spotted wing drosophila, *Drosophila suzukii* Matsumura, is a worldwide pest that originated from Asia and that has infested more than 60 plant species of berries and stone fruits, with economic losses of up to 100% reported in more than 30 countries in Asia, the Americas, and Europe (Adrion et al. 2014, Zhang et al. 2014, Asplen et al. 2015). In general, the drosophila is only harmful to rotten fruits or damaged fruits, while the *D. suzukii* can lay eggs via serrated ovipositor under the skin of healthy ripened fruits. After hatching, the larvae move into the fruit pulp for feeding. Besides this, secondary infection by pathogens often results in fruit rotting, reduction of yield, and loss of marketability (Goodhue et al. 2011, EPPO 2019a). Because of the high potential for spread and the serious economic damage it causes, *D. suzukii* is considered a quarantine pest by the European and Mediterranean Plant Protection Organization (EPPO), Comité de Sanidad Vegetal (COSAVE), the Eurasian Economic Union (EAEU), Jordan, Kazakhstan, Turkey, Morocco, and Mexico (EPPO 2019a, 2019b).

Cold treatment has been studied and widely used as phytosanitary treatment in controlling immature stages of fruit flies and moths in various fresh fruits (Hallman et al. 2013, Jiao et al. 2013, Li et al. 2013, IPPC 2018). However, studies on cold treatment of *D. suzukii* have mainly focused on cold acclimatization and adaptation of adult flies till now. The cold strategy employed by *D. suzukii* is chill-susceptible (Jakobs et al. 2015). Kanzawa first discovered in 1939 that both eggs and larvae of *D. suzukii* in cherries were completely killed when treated at 1.67°C for 96 h (Dalton et al. 2011). In blueberries, no eggs survived to pupation when stored at 1.67°C for 72 h, and survival of third instars was reduced by 41%. In raspberries, egg, second instar, and third instar survival was significantly reduced following storage at 1.67°C for 72 h. There was no egg hatching but reduced third instar survival when stored at 1.1°C for 72 h. The developmental periods of egg and larva in blueberries and raspberries were prolonged after being treated (Aly et al. 2017). Third-instar feeding larvae were more cold-tolerant than third wandering larvae (Jakobs et al. 2017). Some of feeding larvae were still alive after 72-h exposure at 0°C, while wandering larvae all died. However, feeding larvae did not survive longer than 120 h (Jakobs et al. 2017). For phytosanitary cold treatment, Kim et al. (2018) studied the cold tolerance of *D. suzukii* fed on artificial diet. The result of time–response tests suggested that 8-d cold exposure at 1°C could be used for phytosanitary treatment of ‘Campbell Early’ grape.

*Vitis vinifera* L. (both table and wine grapes) are suitable hosts for *D. suzukii* and can be heavily infested in laboratory conditions (Baser et al. 2018, Shrader et al. 2019). The susceptibility of grape varieties to *D. suzukii* oviposition was related to penetrating resistance and Brix (Burrack et al. 2013, Ioriatti et al. 2015, Baser et al. 2018). ‘Red Globe’ grape with lower penetrate force and lower sugar content than other cultivars had certain susceptibility to infestation (Baser et al. 2018). In China, grape is one of the four major fruits, and the quantity of grape exported has gradually increased in recent years (Zhang and Li 2016). However, grape might be infested with *D. suzukii*, which will pose a potential threat to international trade. In addition, although grapes are usually stored and transported at low temperatures (Li et al. 2015, Wang et al. 2016), postharvest cold treatments have never been performed on ‘Red Globe’ grape for quarantine purposes. Therefore, it is necessary to develop phytosanitary cold treatment against *D. suzukii* to promote the export of grapes in China. Thus, the objectives of this study were to find out the most tolerant stage to cold and determine the minimum time required for probit 9 mortality (a mortality of 99.9968%) of *D. suzukii* in ‘Red Globe’ grapes to ensure quarantine security (NAPPO 2011).

## Materials and Methods

### Test Insects

The *D. suzukii* population was collected in an orchard near Yunnan Agricultural University, Kunming, China, from March to April 2017, and newly collected wild adults were introduced into the population once a year. Larvae and adults were reared together in insect cages (30 by 40 by 40 cm) with an artificial diet (wheat bran, sucrose, Brewer’s yeast ≈ 9:3:1) modified from the larvae diet of papaya fruit fly, *Bactrocera papayae* (Liu et al. 2017). All stages were kept in the rearing room at 26 ± 1°C, 50–70% RH with the photoperiod of 14:10 (L:D) h.

### Grape Infestation

Table grapes (cultivar of ‘Red Globe’) were bought from the local market near the Chinese Academy of Inspection and Quarantine, Beijing, China. Pesticides were not applied directly on the fruit and there were no pesticide residues. To prevent microbial contaminations, grapes with similar size, hardness, and color were washed with distilled water, dried at room temperature, and stored in the cold chamber at 0°C. For each time of egg collection, the grapes were taken out and warmed up to room temperature, and a longitudinal cut was made with a sterilized scalpel (Kim et al. 2018), and then the grapes were put into the cage for *D. suzukii* egg laying for 24 h. Every three to four infested grapes (40 ± 5 g for each) were put into one glass jar (0.42 liter) with filter paper at the bottom and covered with gauze to prevent contamination. Each jar was as one replication in the cold treatment and each treatment had three replications (three jars).

### Developmental Time of *D. suzukii* in Grapes

The infested grapes in jars were placed in the rearing room for the development of the *D. suzukii* eggs. When larvae began to pupate, wheat bran was put under the grapes to prevent the larvae and pupae from drowning. Stages were checked every 12 h in the first 2 d and then at an interval of 24 h. At each time point, grapes were removed from three jars and dissected to count the number of each stages under a microscope.

### Cold Tolerance Test

To determine the most tolerant stage against cold, grapes infested with different immature stages based on above rearing experiment of *D. suzukii* were treated at 0°C (0.027 ± 0.168) for 12 h and 2°C (2.034 ± 0.181) for 24 h via three replicates for each stage. Fruit pulp temperatures were recorded every 90 s by a temperature recorder (1586A SUPER-DAQ Precision Temperature Scanner, Fluke Co., WA) with six probes (Pt100, Chong Qing Well Co., Chongqing, China). All the probes were calibrated at the beginning and the end of each trial with a certified mercury glass thermometer. The starting time was recorded as all probes reached the target temperature. Twelve or 24 h later, the infested grapes were transferred to the rearing room for 4 d and the mortality of treated and controlled insects was checked under a microscope. These treatments were repeated three times each.

The criteria for mortality assessment in this research study were as follows: eggs: blacking or unhatched; larvae: no movement when prodded with a blunt probe; pupae: blacking, half-eclosion, deformation of eyes and wings.

### Lethal Time Estimates

The grapes containing 1-d-old eggs, which were defined as the most tolerant stage after the cold tolerance test, were kept into the refrigerator chamber to be cold treated at 0 or 2°C. The core temperatures of the infesting grapes were monitored with six probes. Treatment was deemed to start after all the fruits had reached the target temperature. The grapes were transferred to the rearing room every day till 10-d treatment at 2°C and on the day of 0.5, 1, 1.5, 2, 3, 4, 5, 6, 7, and 8 for 0°C treatment. The mortality of all treatments and controls was checked 4 d later by using the methods mentioned above. The temperature monitored in the treatment was 0.0 ± 0.2 and 2.0 ± 0.2°C (mean ± SE) for the treatment at 0 and 2°C, respectively. These treatments were repeated three times each.

### Confirmatory Test

For each trial of the confirmation test, grapes were put into the adult cages which contained about 2,000 adults from one batch of the colony and kept for 24 h. Then, the infested grapes were put into the cold refrigerate chamber for cold treatment at 0°C for 11 d and 2°C for 12 d according to the result of the efficacy test. The mortality was checked by using the method mentioned above. These treatments were repeated three times at each temperature.

### Statistical Analysis

The mortality data were all adjusted with the Abbott’s formula. The tolerance comparison and time-mortality tests were subject to one-way analyses of variance (ANOVAs) by SPSS (version 17, SPSS Inc., Chicago, IL) (Abbott 1925). Means were compared by Tukey’s test. The time-mortality data of the most tolerant stage were analyzed by using the probit model and logit model (PoloPlus software program 2.0, LeOra Software, Berkeley, CA) to estimate the minimum lethal time for 99% (LT_99_) and 99.9968% mortality (LT_99.9968_) at the 95% confidence level (LeOra Software 2007).

The mortality proportion (1 − *P*_*u*_) of the large-scale confirmatory tests was calculated by the equation (1):

$$\begin{array}{l} 1\;-P_{u}=\;{\left(1\;-\;C\right)}^{1\;/\;n} \end{array}$$ (1)

where *P*_*u*_ is the acceptable level of survivorship, *C* is the confidence level (95%), and *n* is the number of treated *D. suzukii* with zero survivors (Couey and Chew 1986).

## Results

### Developmental Time of *D. suzukii* in Grapes

**Fig. 1.**
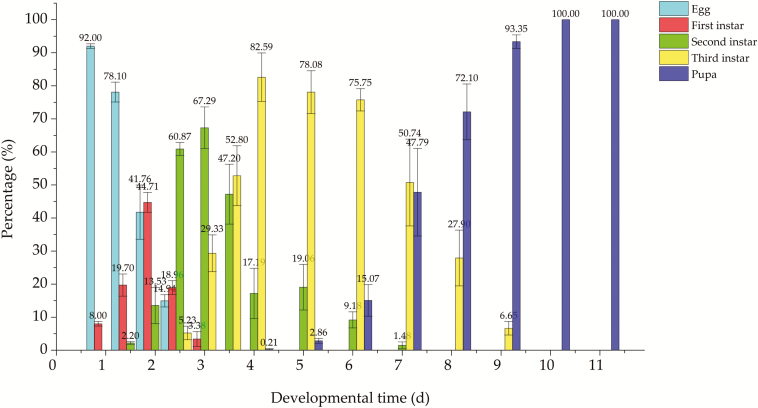
The percentage of immature stages of *Drosophila suzukii* at each developmental time in grapes. Error bars indicated the SE of three independent biological replicates.

The percentage of each immature stage at the specific time was calculated and listed in Fig. 1. The main developmental days (peak time) for egg, first, second, and third instars, and pupa stage were 0–2 (1), 1.5–2.5 (2), 2.5–3.5 (2.5–3), 3.5–7 (4–5), and 7–11 (9–11) days, respectively. The critical time with the similar percentage between stages was 2 d for egg and first instars, 3.5 d for second and third instars, and 7 d for third instars and pupa. Thus, the eggs developed in table grapes for 1, 2, 2.5, 4, 9, and 11 d were considered as the stage of egg, first, second, and third instars, early and late pupa, respectively, to conduct the following time–response test and large-scale confirmatory test. Each growth state observation of immature stage was done in triplication and each replication of eggs, first, second, and third instars included more than 100 test individuals. For pupae, each replication included more than 80 individuals.

### Cold Tolerance Test

The mortality of each stage of *D. suzukii* was significantly different at 0°C for 12 h (*F* = 34.078, df = 5, 12; *P* < 0.001) and 2°C for 24 h (*F* = 36.354, df = 5, 12; *P* < 0.001) (Table 1). The egg stage had the lowest mortality at 0°C (21.49 ± 1.25%) and 2°C (8.98 ± 0.41%) among the different immature stages indicating it was the most tolerant stage to cold. The first instar was the most sensitive (greatest mortality) at (81.46 ± 1.15%) at 2°C, while the second instar was the most sensitive at 0°C (72.65 ± 3.29%). Third instars, and early and late pupa had similar tolerance at 0°C as their developing time overlapped as shown in Fig. 1. Therefore, the sequence from most to least tolerance to 0°C was: egg > third instar, early and late pupa > first and second instars; to 2°C was: egg, third instars > early and late pupa > second and first instars. The 1-d-old egg was selected as the target stage for conducting the following dose–response test and large-scale confirmatory test.

**Table 1.** Mortality of the immature stages of *Drosophila suzukii* in grapes treated at 0°C–12 h and 2°C–24 h

	No. treated		Mortality (%) (mean ± SE)	
Stage (develop time)	0°C–12 h	2°C–24 h	0°C–12 h	2°C–24 h
Egg (1 d)	347	803	21.49 ± 1.25c	8.98 ± 0.41c
First instar (2 d)	455	327	69.68 ± 0.36ab	81.46 ± 1.15a
Second instar (2.5 d)	568	218	72.65 ± 3.29a	61.43 ± 2.24ab
Third instar (4 d)	330	200	54.23 ± 1.24b	24.58 ± 0.61c
Early pupa (9 d)	230	217	57.69 ± 1.15b	45.44 ± 7.19b
Late pupa (11 d)	160	193	67.04 ± 5.93ab	45.68 ± 5.93b

Means within a column followed by different letter a, b, or c were significantly difference (*P* < 0.05; Tukey’s test).

Each growth state observation of immature stage was done in triplication and each replication of eggs and first instar included more than 100 test individuals. For second and third instars and early pupae, each replication included about 70 individuals, while late pupae contained more than 50 individuals.

### Lethal Time Estimates

The mortality of 1-d-old eggs of *D. suzukii* during the time–response test increased significantly with the increase of time when treated at 0°C (*F* = 184.83, df = 10, 22; *P* < 0.001) and 2°C (*F* = 278.85, df = 10, 22; *P* < 0.001) for 8 d. The minimum days to 100% mortality were 6 d at 0°C and 7 d at 2°C, respectively (Table 2). Then, the data were subjected to probit analysis using the probit and logit model. The parameters including slope, heterogeneity (chi-square divided by degrees of freedom), LT_99_, and LT_99.9968_ are presented in Table 3. The models were evaluated based on heterogeneity and LT_99.9968_ by analyzing with the mortality of time–response test. The small value of heterogeneity obtained in the probit model for 0 and 2°C with log-transformed data (2.09–2.95) and the logit model without log-transformed data (2.34–2.73) indicated that the estimations matched the data. Both the LT_99_ and the LT_99.9968_ estimated by probit analysis (with log-transformation) were about 4 d longer than those estimated by the logit model (without log-transformation). The heterogeneity data in these two models were similar. Therefore, 7- and 11-d cold treatment at 0°C, and 9- and 12-d cold treatment at 2°C were all selected as the target lethal time for conducting the following confirmatory large-scale test (Table 4).

**Table 2.** Mortality of 1-d-old eggs, the most cold-tolerant developmental stage of *Drosophila suzukii* treated at 0 and 2°C in grapes

	Treatment at 0°C		Treatment at 2°C	
Exposure time (d)	No. treated	Mortality (%) (mean ± SE)	No. treated	Mortality (%) (mean ± SE)
0 (control)	1,184	10.93 ± 1.37d	1,184	10.93 ± 1.37d
0.5	409	16.27 ± 6.03d	–	–
1	564	33.26 ± 1.42c	362	8.72 ± 1.15d
1.5	436	73.37 ± 4.81b	–	–
2	832	82.27 ± 3.38b	347	46.17 ± 4.16c
3	392	96.96 ± 1.32a	318	81.99 ± 4.94b
4	388	99.31 ± 0.34a	395	94.46 ± 2.26a
5	543	99.80 ± 0.20a	301	96.07 ± 1.55a
6	404	100.00 ± 0.00a	586	99.78 ± 0.23a
7	396	100.00 ± 0.00a	534	100.00 ± 0.00a
8	733	100.00 ± 0.00a	462	100.00 ± 0.00a

Means within a column followed by different letter a, b, or c were significantly difference (*P* < 0.05; Tukey’s test).

**Table 3.** Estimation of the minimum lethal time of the 1-d-old eggs of *Drosophila suzukii* treated at 0 and 2°C in grapes

Temp. (°C)	Analyzing model (log-transformed)	Slope ± SE	LT_99_ (d) (95% CI)	LT_99.9968_ (d) (95% CI)	Heterogeneity
0	probit (with)	4.20 ± 0.19	4.18 (3.65–5.02)	10.47 (8.10–14.92)	2.95
	probit (without)	1.13 ± 0.05	3.28 (2.70–4.68)	4.76 (3.75–7.27)	24.44
	logit (with)	7.77 ± 0.37	4.67 (3.95–5.90)	25.74 (16.95–46.74)	3.33
	logit (without)	2.05 ± 0.09	3.47 (3.06–4.12)	6.28 (5.34–7.80)	2.73
2	probit (with)	5.23 ± 0.28	5.70 (5.14–6.55)	11.92 (9.78–15.64)	2.09
	probit (without)	0.82 ± 0.04	5.02 (4.62–5.56)	7.05 (6.38–8.00)	3.35
	logit (with)	9.89 ± 0.59	6.10 (5.33–7.40)	23.35 (16.62–38.35)	2.30
	logit (without)	1.53 ± 0.08	5.18 (4.78–5.72)	8.96 (8.07–10.21)	2.34

**Table 4.** Large-scale cold treatment trials with the eggs of *Drosophila suzukii* in grapes at 0 and 2°C

No. of trials	Treatment	No. of fruits	Estimated no. of treated eggs	No. of living larvae
1	2°C–9 d	700	24,500	8
	Control	40	–	1,466
2	0°C–11 d	480	18,697	0
	2°C–12 d	528	20,567	0
	Control	42	–	1,636
3	0°C–11 d	480	16,973	0
	2°C–12 d	539	19,060	0
	Control	36	–	1,273
4	0°C–11 d	480	21,696	0
	2°C–12 d	238	10,758	0
	Control	40	–	1,808

### Confirmatory Test

Confirmatory tests were conducted to validate the estimated time to 99.9968% mortality of the 1-d-old eggs (the most tolerant stage in harvested grapes) of *D. suzukii*. For each trial, 238–539 grapes could collect more than 20,000 eggs. The temperature monitored during treatments was at 0.0 ± 0.2 or 2.0 ± 0.2°C (mean ± SE), respectively. For the treatment at 2°C for 9 d, eight living larvae were found in the treated grapes containing more than 24,500 eggs in the first trial, suggesting that the logit model was not appropriate. However, there were no survivors from a total of 57,366 eggs and 50,385 eggs treated at 0°C for 11 d and 2°C for 12 d, respectively (Table 4), indicating the probit model was validated. The disinfestation efficacy was 99.9948 and 99.9941% at 0 and 2°C, respectively.

## Discussion

Cold treatment, which is considered a common method to eliminate pests in fresh fruits and other products, has been studied, analyzed, and incorporated into quarantine regulations for a number of years (Jessup et al. 1993, De Lima et al. 2011, Grout et al. 2011, Dohino et al. 2017, Fang et al. 2019). To develop the phytosanitary measure, the most tolerant stage should be compared and subjected to dose–response test and confirmatory test (IPPC 2007). Generally, pests in their natural habitat are optimal for the tolerance test (NAPPO 2011). In this study, developmental duration of the immature stages of *D. suzukii* was firstly observed in ‘Red Globe’ grapes to select the appropriate stages used in the cold tolerance test (Fig. 1). To obtain high levels of oviposition in grapes, a cut with a scalpel was made on the surface of ‘Red Globe’, according to the result of Shrader et al. (2019) that more flies emerging from injured grapes than intact grapes. Based on mortality estimates, the 1-d-old egg appeared to be the most cold-tolerant stage with the lowest mortality among different developing stages, both at 0 and 2°C (Table 1). The results are consistent with research on the model drosophila fruit fly, *Drosophila melanogaster* Meigen, whose eggs were the most tolerant in cold shock tests by assaying LT_50_ over −16 to 5°C for 1 h, followed by adults, pupae, and larvae (Jensena et al. 2007). However, Kim et al. (2018) reported that pupal stage of *D. suzukii* was more resistant than egg, and it was defined as the most tolerant stage with lab diet. Besides, to achieve complete mortality of *D. suzukii* immature stages reared on an artificial diet, the exposure durations required were 6 d at 1°C, and 8 d at 1.5 and 2°C (Kim et al. 2018). However, in our test, a few larvae emerged from the treatment at 2°C for 9 d. The reasons why our conclusions were not the same might be different infestation method, feeding conditions, temperature, fruit type, colony source, and the choice of mortality endpoint. In the present study, in order to get closer to the real-world environment, we chose to study cold response from host fruit of *D. suzukii*, rather than the lab diet, which might resulted in differences in fly physiology and development at low temperature. Furthermore, each stage used in cold-tolerate trial was actually a composite of multiple developmental stages, for example, 1-d-old egg containing approximately 92.0% egg and 8.0% first instars, early pupa containing 6.7% third instars, and 93.4% pupa. This condition was similar to the cold tolerance test of the Mediterranean fruit fly, *Ceratitis capitata* (Wiedemann), whose mixed stages were treated in host fruit (Armstrong et al. 1995, De Lima et al. 2011, Ware and Du Toit 2017). This could also lead to the different results of cold response in the same species.

Usually, the probit model and logit model are used to estimate the minimal lethal time of 99.9968% mortality according to the time–response test (Follett et al. 2018, Al-Behadili et al. 2019, Fang et al. 2019). In the present study, four different models were selected separately for eggs at every temperature. Log on days with probit and no-log on days with logit were selected to estimate the LT_99_ and LT_99.9968_ as the best fitting models (Table 3). There was no survivor from a total of more than 50,000 eggs treated at 0°C for 11 d and 2°C for 12 d, respectively (Table 4). However, eight larvae emerged from the confirmatory tests when treated at 2°C for 9 d (based on the estimation of the logit model). The result suggested that the probit model was more suitable for analyzing the cold treatment of *D. suzukii*, which was similar to cold treatment of the *C. capitata* (Wiedemann), *Bactrocera tryoni* (Froggatt), *B. dorsalis* (Hendel), and *B. zonata* (Saunders) (De Lima et al. 2007, Hallman et al. 2013, Myers et al. 2016). In addition, the minimal time for probit 9 mortality tested in the large-scale confirmatory tests may also be affected by the host fruit, cold tolerance stage, endpoint checking and infesting method.

Besides the disinfestation of the infesting insects in the commodity, there should be no significant deleterious effects on the quality of the fruit. In general, the ‘Red Globe’ grape could be stored at an optimum temperature of −1 to 0°C to keep the quality for more than 1 month (Wu 2015). Several research studies have confirmed that a 12-d cold treatment at 0 and 1°C caused no significant damage to the quality of grapes (Wuernisha et al. 2010), and there is no significant negative effect on the taste of ‘Red Globe’ grape treatment at 0 and 2°C for 14 d in the primary test. Therefore, the schedules developed from this research could be used for the phytosanitary treatment of table grape ‘Red Globe’.

### Conclusions

In this research, the effects of phytosanitary cold treatment of *D. suzukii* in ‘Red Globe’ grape were examined. The results show that among six developmental stages (egg, first, second, and third instars, early and late pupa) of *D. suzukii* in grapes, egg was the most cold-tolerant stage. The time–response test and large-scale confirmatory test suggest that a minimum of 11-d cold treatment at 0°C and 12-d at 2°C were recommended for disinfesting *D. suzukii* in grapes with efficacy of 99.9941 and 99.9948%, in order to provide postharvest pest control and quarantine security for international trade. All results suggest that cold treatment could be used as a methyl bromide alternative for quarantine control of *D. suzukii* in ‘Red Globe’ grape.

## Notes

The Chinese Academy of Inspection and Quarantine and the China Agricultural University also contributed equally. Correspondence and requests for materials should be addressed to Bo Liu (liub@caiq.org.cn) and Zhihong Li (lizh.ento@263.net).

## References

[CIT0001] Abbott W. S 1925 A method of computing the effectiveness of an insecticide. J. Econ. Entomol. 18: 265–267.

[CIT0002] Adrion J. R., Kousathanas A., Pascual M., Burrack H. J., Haddad N. M., Bergland A. O., Machado H., Sackton T. B., Schlenke T. A., Watada M., et al 2014 *Drosophila suzukii:* the genetic footprint of a recent, worldwide invasion. Mol. Biol. Evol. 31: 3148–3163. 25158796 10.1093/molbev/msu246 PMC4245814

[CIT0003] Al-Behadili F. J. M., Bilgi V., Li J., Wang P., Taniguchi M., Agarwal M., Ren Y., and Xu W.. 2019 Cold response of the Mediterranean fruit fly (*Ceratitis capitata*) on a lab diet. Insects 10: 48. 10.3390/insects10020048 PMC6409936 30717472

[CIT0004] Aly M. F., Kraus D. A., and Burrack H. J.. 2017 Effects of postharvest cold storage on the development and survival of immature *Drosophila suzukii* (Diptera: Drosophilidae) in Artificial diet and fruit. J. Econ. Entomol. 110: 87–93. 28039427 10.1093/jee/tow289

[CIT0005] Armstrong J. W., Silva S. S., and Shishido V.. 1995 Quarantine cold treatment for Hawaiian carambola fruit infested with Mediterranean fruit fly, melon fly, or oriental fruit fly (Diptera: Tephritidae) eggs or larvae. J. Econ. Entomol. 88: 683–687.

[CIT0006] Asplen M. K., Anfora G., Biondi A., Choi D. S., Chu D., Daane K. M., Gibert P., Gutierrez A. P., Hoelmer K. A., Hutchison W. D., et al 2015 Invasion biology of spotted wing drosophila (*Drosophila suzukii*): a global perspective and future priorities. J. Pest Sci. 88: 469–494.

[CIT0007] Baser N., Broutou O., Verrastro V., Porcelli F., Ioriatti C., Anfora G., Mazzoin V., and Rossi Staccoin M. V.. 2018 Susceptibility of table grape varieties grown in south-eastern Italy to *Drosophila suzukii*. J. Appl. Entomol. 142: 465–472.

[CIT0008] Burrack H. J., Fernandez G. E., Spivey T., and Kraus D. A.. 2013 Variation in selection and utilization of host crops in the field and laboratory by *Drosophila suzukii* Matsumara (Diptera: Drosophilidae), an invasive frugivore. Pest Manag. Sci. 69: 1173–1180. 23494939 10.1002/ps.3489

[CIT0009] Couey H. M., and Chew V.. 1986 Confidence limits and sample size in quarantine research. J. Econ. Entomol. 79: 887–890.

[CIT0010] Dalton D. T., Walton V. M., Shearer P. W., Walsh D. B., Caprile J., and Isaacs R.. 2011 Laboratory survival of *Drosophila suzukii* under simulated winter conditions of the Pacific Northwest and seasonal field trapping in five primary regions of small and stone fruit production in the United States. Pest Manag. Sci. 67: 1368–1374. 22021034 10.1002/ps.2280

[CIT0011] De Lima C. P. F., Jessup A. J., Cruickshank L., Walsh C. J., and Mansfield E. R.. 2007 Cold disinfestation of citrus (*Citrus* spp.) for Mediterranean fruit fly (*Ceratitis capitata*) and Queensland fruit fly (*Bactrocera tryoni*) (Diptera: Tephritidae). N. Z. J. Exp. Agric. 35: 39–50.

[CIT0012] De Lima C. P. F., Jessup A. J., Mansfield E. R., and Daniels D.. 2011 Cold treatment of table grapes infested with Mediterranean fruit fly *Ceratitis capitata* (Wiedemann) and Queensland fruit fly *Bactrocera tryoni* (Froggatt) Diptera: Tephritidae. N. Z. J. Crop Hortic. Sci. 39: 95–105.

[CIT0013] Dohino T., Hallman G. J., Grout T. G., Clarke A. R., Follett P. A., Cugala D. R., and Myers S. W.. 2017 Phytosanitary treatments against *Bactrocera dorsalis* (Diptera: Tephritidae): current situation and future prospects. J. Econ. Entomol. 110: 67–79. 28028169 10.1093/jee/tow247

[CIT0014] European and Mediterranean Plant Protection Organization (EPPO) 2019a Standard PM 1/2 (28): EPPO A1 and A2 lists of pests recommended for regulation as quarantine pests. EPPO, Paris, France.

[CIT0015] European and Mediterranean Plant Protection Organization (EPPO) 2019b Drosophila suzukii (DROSUU). EPPO, Paris, France.

[CIT0016] Fang Y., Kang F., Zhan G., Ma C., Li Y., Wang L., Wei Y., Gao X., Li Z., and Wang Y.. 2019 The effects of a cold disinfestation on *Bactrocera dorsalis* survival and navel orange quality. Insects 10: 452. 10.3390/insects10120452 PMC6955761 31847197

[CIT0017] Follett P. A., Manoukis N. C., and Mackey B.. 2018 Comparative cold tolerance in *Ceratitis capitata* and *Zeugodacus cucurbitae* (Diptera: Tephritidae). J. Econ. Entomol. 111: 2632–2636. 30085183 10.1093/jee/toy227

[CIT0018] Goodhue R. E., Bolda M., Farnsworth D., Williams J. C., and Zalom F. G.. 2011 Spotted wing drosophila infestation of California strawberries and raspberries: economic analysis of potential revenue losses and control costs. Pest Manag. Sci. 67: 1396–1402. 21815244 10.1002/ps.2259

[CIT0019] Grout T. G., Stephen P. R., Daneel J. H., and Hatfingh V.. 2011 Cold treatment of *Ceratitis capitata* (Diptera: Tephritidae) in oranges using a larval endpoint. J. Econ. Entomol. 104: 1174–1179. 21882680 10.1603/ec10434

[CIT0020] Hallman G. J., Myers S. W., Taret G., Fontenot E. A., and Vreysen M. J. B.. 2013 Phytosanitary cold treatment for oranges infested with *Bactrocera zonata*. J. Econ. Entomol. 106: 2336–2340. 24498731 10.1603/ec13221

[CIT0021] International Plant Protection Convention (IPPC) 2007 ISPM 28: phytosanitary treatments for regulated pests. FAO, Rome, Italy.

[CIT0022] International Plant Protection Convention (IPPC) 2018 ISPM 42: requirements for the use of temperature treatments as phytosanitary measures. FAO, Rome, Italy.

[CIT0023] Ioriatti C., Walton V., Dalton D., Anfora G., Grassi A., Maistri S., and Mazzoni V.. 2015 *Drosophila suzukii* (Diptera: Drosophilidae) and its potential impact to wine grapes during harvest in two cool climate wine grape production regions. J. Econ. Entomol. 108: 1148–1155. 26470240 10.1093/jee/tov042

[CIT0024] Jakobs R., Gariepy T. D., and Sinclair B. J.. 2015 Adult plasticity of cold tolerance in a continental-temperate population of *Drosophila suzukii*. J. Insect Physiol. 79: 1–9. 25982520 10.1016/j.jinsphys.2015.05.003

[CIT0025] Jakobs R., Ahmadi B., Houben S., Gariepy T. D., and Sinclair B. J.. 2017 Cold tolerance of third-instar *Drosophila suzukii* larvae. J. Insect Physiol. 96: 45–52. 27765625 10.1016/j.jinsphys.2016.10.008

[CIT0026] Jensena D., Overgaarda J., and Sørensenb J. G.. 2007 The influence of developmental stage on cold shock resistance and ability to cold-harden in *Drosophila melanogaster*. J. Insect Physiol. 53: 179–186. 17234205 10.1016/j.jinsphys.2006.11.008

[CIT0027] Jessup A. J., Delima C. P. F., Hood C. W., Sloggett R. F., Harris A. M., and Beckingham M.. 1993 Quarantine disinfestation of lemons against *Bactrocera tryoni* and *Ceratitis capitata* (Diptera, Tephritidae) using cold-storage. J. Econ. Entomol. 86: 798–802.

[CIT0028] Jiao S., Johnson J. A., Tang J., Mattinson D. S., Fellman J. K., Davenport T. L., and Wang S.. 2013 Tolerance of codling moth, and apple quality associated with low pressure/low temperature treatments. Postharvest Biol. Technol. 85: 136–140.

[CIT0029] Kim M. J., Kim J. S., Jeong J. S., Choi D. S., Park J., and Kim I.. 2018 Phytosanitary cold treatment of spotted-wing drosophila, *Drosophila suzukii* (Diptera: Drosophilidae) in ‘Campbell Early’ grape. J. Econ. Entomol. 111: 1638–1643. 29850850 10.1093/jee/toy148

[CIT0030] LeOra Software 2007 PoloPlus user’s guide, version 2nd ed. LeOra Software, Berkeley, CA.

[CIT0031] Li Z., Jiang F., Ma X., Fang Y., Sun Z., Qin Y., and Wang Q.. 2013 Review on prevention and control techniques of Tephritidae invasion (in Chinese). Plant Quar. 27: 1–10.

[CIT0032] Li Z., Ha Y., Li Y., Li Q., and Jin J.. 2015 Effects of different treatment on quality of ‘Red Globe’ grape during refrigeration (in Chinese). J. Chin. Institute Food Sci. Technol. 15: 123–128.

[CIT0033] Liu B., Li B., Zhan G., Zha T., Wang Y., and Ma C.. 2017 Forced hot-air treatment against *Bactrocera papayae* (Diptera: Tephritidae) in papaya. Appl. Entomol. Zool. 52: 531–541.

[CIT0034] Myers S. W., Cancio-Martinez E., Hallman G. J., Fontenot E. A., and Vreysen M. J. B.. 2016 Relative tolerance of six *Bactrocera* (Diptera: Tephritidae) species to phytosanitary cold treatment. J. Econ. Entomol. 109: 2341–2347. 27660425 10.1093/jee/tow206

[CIT0035] North American Plant Protection Organization (NAPPO). 2011 NAPPO Regional Standards for Phytosanitary Measures (RSPM) 34: development of phytosanitary treatment protocols for regulated arthropod pests of fresh fruits or vegetables. NAPPO, Ottawa, ON, Canada.

[CIT0036] Shrader M. E., Burrack H. J., and Pfeiffer D. G.. 2019 *Drosophila suzukii* (Diptera: Drosophilidae) oviposition and adult emergence in six wine grape varieties grown in Virginia. J. Econ. Entomol. 112: 139–148. 30407506 10.1093/jee/toy305

[CIT0037] Wang X., Wang J., Liu X., Du F., and Guan X.. 2016 Effect of different preservation treatment on storage in low temperature of ‘Red Globe’ grape (in Chinese). Food Ind. 37: 98–102.

[CIT0038] Ware A. B., and du Toit C. L. N.. 2017 Cold disinfestation of “Hass” avocado (*Persia americana*) of three species of fruit fly (Diptera: Tephritidae)-*Ceratitis capitata*, *Ceratitis rosa*, and *Ceratitis cosyra*. J. Econ. Entomol. 110: 954–960. 28444314 10.1093/jee/tox068

[CIT0039] Wu S 2015 Different effects of pre-cooling methods on ‘Red Globe’ grape (in Chinese). China Fruit Vegetable 8: 1–3.

[CIT0040] Wuernisha K., Che B., Zhang T., Li P., and Hu B.. 2010 Effect of different temperature on quality and physiological index of postharvest ‘Red Globe’ grape during storage (in Chinese). Xinjiang Agric. Sci. 47: 82–86.

[CIT0041] Zhang J., and Li P.. 2016 Research progress of postharvest preservation technology on ‘Red Globe’ grape (in Chinese). North. Hortic. 10: 181–184.

[CIT0042] Zhang K., Yan G., Guo X., Wang J., Zhang X., and Zhou Y.. 2014 Research review on spotted wing drosophila (*Drosophila suzukii*) (in Chinese). J. Fruit Sci. 31: 717–721.

